# Greener Solutions in Aflatoxin Management: Transitioning from Conventional Binders to Green Nanotechnology

**DOI:** 10.3390/nano15211604

**Published:** 2025-10-22

**Authors:** Patience M. Awafong, Viola O. Okechukwu, Temitope R. Fagbohun, Oluwasola A. Adelusi, Oluwafemi A. Adebo, Patrick B. Njobeh, Julian Q. Mthombeni

**Affiliations:** 1 Department of Biomedical Sciences, Faculty of Health Sciences, University of Johannesburg, Doornfontein Campus, Johannesburg P.O. Box 17011, South Africa; patiencemaghah85@gmail.com; 2 Department of Environmental Water and Earth Sciences, Faculty of Science, Tshwane University of Technology, Pretoria 0001, South Africa; violaokafor15@gmail.com; 3 Department of Human Anatomy and Physiology, Faculty of Health Sciences, University of Johannesburg, Doornfontein Campus, Johannesburg P.O. Box 17011, South Africa; temitopesms2@gmail.com; 4 Department of Biotechnology and Food Technology, Faculty of Science, University of Johannesburg, Doornfontein Campus, Johannesburg P.O. Box 17011, South Africa; solaceonline2009@gmail.com; 5 Centre for Innovative Food Research (CIFR), Department of Biotechnology and Food Technology, Faculty of Science, University of Johannesburg, Doornfontein Campus, Johannesburg P.O. Box 17011, South Africa; oadebo@uj.ac.za

**Keywords:** aflatoxins, mitigation, toxin binders, green nanotechnology, β-cyclodextrin

## Abstract

Aflatoxins (AFs) are toxic metabolites produced by *Aspergillus flavus* (*A. flavus*) and *Aspergillus parasiticus* (*A. parasiticus*) that contaminate food and feed, posing serious health risks to humans and animals. Consumption of aflatoxin-contaminated foods can cause aflatoxicosis, a serious condition characterised by acute or chronic toxicity. Due to their prevalence, especially in humid regions such as sub-Saharan Africa, proper management is essential for food safety and public health. While traditional methods for controlling aflatoxins can be effective, they are often costly and may introduce harmful chemicals into food, posing risks to the environment and human health. This review paper extensively analyses the toxin binders used to mitigate aflatoxin contamination, discussing their mechanisms of action and effectiveness. It also explores the transition from traditional aflatoxin management strategies to greener alternatives, with a focus on the emerging field of green nanotechnology. Additionally, this paper examines the biosynthesis of nanoparticles (NPs) using metal salt solutions and plant extracts, and their efficacy as inhibitors of aflatoxin-producing fungi and their toxins, demonstrating high effectiveness with minimal toxicity to human health and the environment. Furthermore, the article explores the integration of green nanotechnology into sustainable aflatoxin management and discusses future research directions for developing even more potent interventions through nano-encapsulation with β-cyclodextrin (β-CD).

## 1. Introduction

Aflatoxins (AFs) are a collection of chemically related mycotoxins originating from toxigenic strains of fungi, primarily from the genera *Aspergillus flavus* (*A. flavus*) and *Aspergillus parasiticus* (*A. parasiticus*) [1,2,3]. *Aspergillus*, the fungus responsible for aflatoxin production, is commonly found in various natural environments. They thrive on several organic nutrient sources, like dead plant material, cotton, animal feed, decaying wood, compost piles, tree leaves, dead insects, animal carcasses, and stored grains. This fungus thrives in warm temperatures, ideally between 24 °C and 35 °C, and humid conditions where moisture contents exceed 7%, such as in tropical and sub-tropical countries lying between latitudes 40 °N and 40 °S of the equator [4,5]. Globally, approximately 4.5 billion people are inadvertently exposed to AFs through their diet [6,7,8]. Aflatoxins are often found contaminating numerous agricultural commodities, with a particular affinity for cereals, nuts and grains such as rice, soybean, maize, peanuts, walnuts, and occasionally in milk, cheese, fruits, spices and cottonseed [3,9].

Aflatoxins gained scientific prominence in the 1960s due to their association with the turkey “X” disease epidemic [10,11] This outbreak was characterised by severe hepatic disease, resulting in high mortality rates in chickens, ducks, and turkeys, fed mold-contaminated feed [9]. This incident brought AFs to the forefront of scientific research and regulatory attention, highlighting the need to better understand their production, prevalence, and impact on human and animal health.

**Figure 1:**
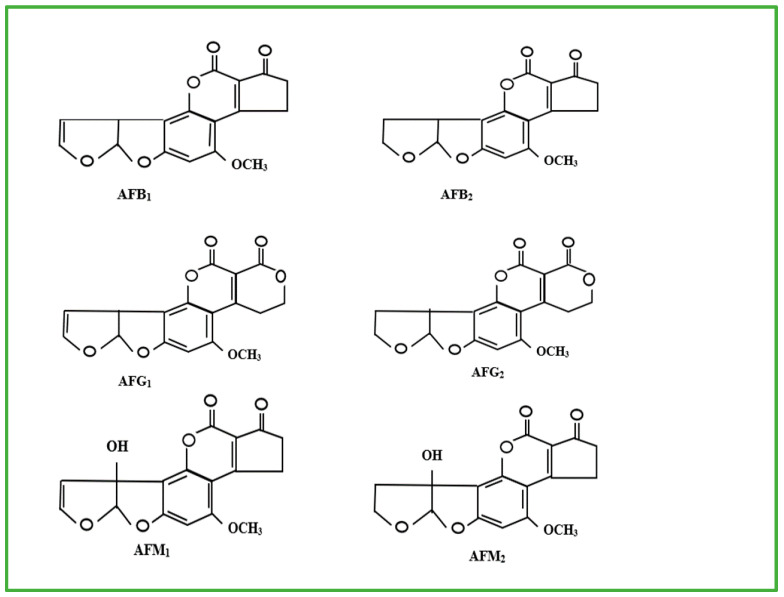
Molecular structures of AFB_1_, AFB_2_, AFG_1_, AFG_2_, AFM_1_ and AFM_2_ [16].

So far, about twenty different AFs have been recognised [12,13]. The most prevalent and significant AFs (Figure 1) are aflatoxin B_1_ (AFB_1_), aflatoxin B_2_ (AFB_2_), aflatoxin G_1_ (AFG_1_), and aflatoxin G_2_ (AFG_2_), as well as aflatoxin M_1_ (AFM_1_) and aflatoxin M_2_ (AFM_2_) [14]. Aflatoxins B_1_, B_2_, G_1_, and G_2_ are naturally produced by *A. flavus* and *A. parasiticus* [3,15]. The aflatoxins M_1_ and M_2_ on the other hand, are metabolites of AFB_1_ found in dairy produce from cows fed with AFB_1_-contaminated feed_._

**Figure 2:**
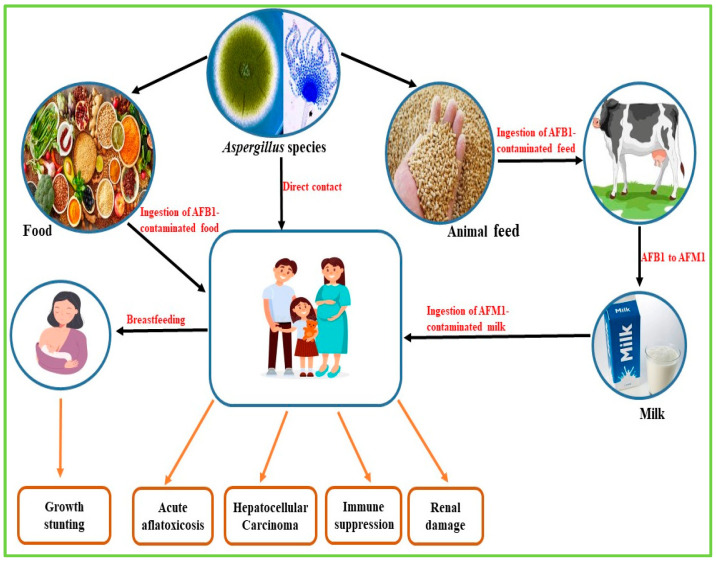
Routes of contamination and health implications of aflatoxin exposure in humans.

Aflatoxin contamination occurs in the field where susceptible food crops become infected by aflatoxigenic fungi, during the post-harvest transportation of food crops and also during storage [17]. AF contamination is estimated to cause the loss of approximately 25% of global food crops annually, resulting in severe economic loss [18]. Exposure to AFs is a common phenomenon mostly in sub-Saharan African countries where adequate control measures are lacking, beginning as early as intrauterine, through the ingestion of contaminated food crops during pregnancy [19,20]. This could persist throughout the individual’s lifespan, resulting in growth stunting, hepatocellular carcinoma, aflatoxicosis and kidney damage in humans (Figure 2). At the same time, in animals, conditions such as fetal death, low birth weight, bone and visceral deformities, decreased immune capacity, and behavioural abnormalities have been observed [19].

Current AF management strategies focus on preventing fungal infection through good agricultural practices, improved storage, and biological, physical, and chemical control measures. If contamination does persist, post-harvest decontamination or detoxification measures are required to reduce the impact of AF contamination. Binding agents are commonly added to AF-contaminated food and feed, reducing or preventing exposure to these toxins. These binding agents (toxin binders/degraders) form a strong bond with AFs in the contaminated food, which remains stable as it passes through the gastrointestinal tract (GIT). The toxin–binder complex is then safely excreted, limiting the effects of AFs on human and animal health.

Conventional aflatoxin management strategies often face limitations such as environmental harm, potential toxicity, and nutrient losses in treated food products [21]. In response to these challenges, green nanotechnology has emerged as a promising and environmentally sustainable alternative. By harnessing naturally derived materials and eco-friendly synthesis methods, green nanomaterials offer innovative solutions that effectively inhibit aflatoxin-producing fungi and degrade toxins with minimal environmental impact [22].

## 2. Mitigating Aflatoxin Exposure Using Toxin Binders

One method for minimising exposure to AFs is to reduce their absorption into cells by incorporating various toxin-binding agents in food and feed products, thereby preventing them from reaching critical organs such as the liver. Toxin binders or absorbents are substances that can absorb, bind, or neutralise toxins, and help eliminate them from the body through stool or bile [23]. Their ring-like structures, and tetrahedral shapes, containing pores with electrical charges, give them the ability to bind AFs [24,25], attracting them through a negative charge in the body, trapping them or adhering to bile that packages the toxins. This process facilitates the elimination of toxins through bowel movements, preventing them from being reabsorbed. This reduces the absorption and bioavailability of toxins in the circulatory system. Several toxin binders are commercially available for use in various medical and health contexts to manage poisoning, detoxification, and related conditions (Table 1). The most widely researched toxin binders have been applied in the food industry to control mycotoxicosis which arises from consuming feeds and food commodities contaminated by mycotoxins. Gruber-Dorninger et al. [26] reported that 88% of feed samples evaluated globally contained at least one or more mycotoxins.

### 2.1. Inorganic and Organic Toxin Binders

#### 2.1.1. Inorganic Binders

##### Activated Carbon or Charcoal

Activated carbon (AC) is a fine black powder produced by charring any material containing carbon, such as wood, coal, or bamboo. The activation process involves a chemical treatment step in which the carbon compound is infused with chemical substances such as phosphoric acid, zinc chloride, or potassium hypochlorite, and exposed to temperatures of 250 °C–600 °C. This is followed by a physical treatment in which the carbon compound is passed through a heated chamber (600 °C–900 °C) with oxygen (O_2_) carbon dioxide (CO_2_) flow. The resulting carbon compound is a highly microporous AC with a high surface area (500–3000 m^2^/g) [27]. Activated carbon has been recommended and is routinely used as a general toxin-binding agent in several digestive toxicities and is beneficial in firming loose stools, thereby addressing gastrointestinal issues [28]. Also, AC has been shown to bind toxins released during food poisoning, thereby reducing the severity of symptoms [29]. Studies have shown that AC effectively binds mycotoxins in foodstuffs and the human body. Specifically, AC products have been shown to bind zearalenone (ZEN) and ochratoxin A (OTA) with high efficiency. Controversies exist in the classification of AC; while some researchers classify it as an inorganic binder [25], others have classified it as an organic binder [30]. The dual classification of activated carbon stems from its origin and its properties. Activated carbon is produced from organic materials such as wood, coconut shells, or coal, which is why it is sometimes loosely grouped with organic binders [31]. However, after activation, it contains no living organic content and, both functionally and chemically, behaves like an inorganic material. Because of its inert, mineral-like adsorption properties, AC is frequently classified as an inorganic binder in practical applications [25].

One of the earliest studies on mycotoxin binding using activated carbon was conducted by Hatch et al. [32], demonstrating its effectiveness in reducing aflatoxicosis in goats. Another study by Obaid, Al-Warshan and Abed [33] on the capacity of AC to lower the adverse effects of AFB_1_ in contaminated broiler diet suggested that adding AC to broiler feed substantially reduced the adverse effects of AFB_1_ on broiler health. These findings align with those reported by Ahn et al. [34] in their in vitro study evaluating the effectiveness of various mycotoxin binders against ZEN, deoxynivalenol (DON), and AFB_1_. Amongst the binders tested, AC was the only binder that was able to effectively absorb all three mycotoxins. The high sequestering ability of AC is due to its large surface area and pore size. Though AC can effectively sequester toxins, it has some disadvantages in its use. It does not aid in repairing a damaged gut lining resulting from toxin exposure or infections. For certain individuals, its taste may be unpalatable, and some may suffer from adverse side effects, like constipation and nausea.

##### Silicates (Clay)

Silicates or clays are made up of silicon dioxide (SiO_2_), commonly known as quartz, which is a key component of the Earth’s crust. When SiO_2_ combines with other substances, silicates are formed, which are potent adsorbents. Aluminosilicates are formed when SiO_2_ bonds with native Aluminium. They are further subdivided into many groups based on their structures: the phyllosilicates (silicate sheets), the tectosilicates (silicate framework), among others [35]. Bentonites, montmorillonites, smectites, illites and kaolinite are phyllosilicates [30] while zeolite and clinoptilolite are tectosilicates [36]. Amongst these two, the phyllosilicates are the most widely researched category. Clays have been recognised as the most efficient adsorbents because of their large specific surface area, hydrophilic surface, pore volume, and negative surface charge [37].

Many clays have been tested and confirmed to be great toxin binders and are capable of reducing the toxic load before consumption as well as in the gastrointestinal tract (GIT). Maged et al. [37] evaluated the potential of natural bentonite collected from Egypt to remove residual ciprofloxacin from water. The results revealed an increased absorption of ciprofloxacin from water from 126.56 to 305.20 mg/g with an enhanced sorption capacity upon acid activation of the bentonite clay. A similar study, conducted by Zabiulla et al. [38], examined the effectiveness of a smectite-based clay binder, Toxo-MX, in mitigating the toxic effects of AFB_1_ in commercial broiler chickens. The results showed that supplementing a diet containing AFB_1_ with this smectite-based mycotoxin binder improved growth performance, decreased liver toxicity, and strengthened the humoral immune response in broilers, demonstrating its protective effect against aflatoxicosis. Wang et al. [39], investigated the ability of sodium montmorillonite to bind Microcystins (MCs) under conditions simulating an in vitro gastrointestinal tract. The results indicated that edible montmorillonites are both safe and effective microcystin (MC) binders. Furthermore, incorporating them into meals throughout algal bloom seasons may help protect susceptible populations of both animals and humans. In a study by Wang et al. (2019) [40], calcium montmorillonite clay (NovaSil) was found to effectively bind AFB_1_ and alleviate the symptoms of aflatoxicosis in animals. To enhance the effectiveness of clay-based sorbents, they developed calcium and sodium montmorillonite clays infused with nutrients such as choline and L-carnitine, which enhanced their sorption capacity for AFB_1_ and provided significant protection against AFB_1_ toxicity in adult hydra, even at low inclusion rates. The study revealed that AFB_1_ primarily adsorbs within the clay interlayer, indicating that the modified clays could be good enterosorbents for screening hazardous chemicals in food and feed during AF contamination outbreaks [40].

Zeolite binders bind toxins ingested through food and those excreted by the liver in bile, thereby preventing the recycling of toxins from the gut by blocking enterohepatic recirculation. Research has shown that natural zeolite-clinoptilolite can adsorb AFs and other mycotoxins, including fumonisins. However, modified zeolites are more potent at absorbing fumonisins compared to natural ones [30]. On the other hand, certain clays (like bentonites) may interfere with the uptake of some minerals like iron, magnesium, zinc, calcium, iodine, selenium, and vitamins, which are essential minerals required for proper growth and development [41], and may cause intestinal cell damage in large doses [42].

#### 2.1.2. Organic Binders

##### Yeast Cell Walls (YCW)

These are primary organic binders used to reduce the toxicity of mycotoxins. *Saccharomyces cerevisiae*’s cell wall is made of lipids, proteins, and polysaccharides such as glucan, mannan, and chitin. Recently, yeast and its derivatives have been employed as feed additives, providing health benefits for farm animals. The main structural component of YCW is a polysaccharide cell wall, which constitutes 90% of the cell mass and gives it its shape. It is made up of two sheets; the outer sheet is a smooth membrane and consists of glucomannans and mannoproteins. This outer sheet determines the superficial properties of the cell wall. The inner sheet is composed of β-(1,3)-d-glucans helix chains organised in a complex three-dimensional structure, and β-(1,6)-d-glucans linear side chains. It confers rigidity and influences the morphology of the yeast [25]. Beta-d-glucans (β-d-glucans) are components of the yeast, responsible for mycotoxin complexation. The efficacy of this complexation is significantly influenced by the reticular configuration of β-d-glucans and the balance between β-(1,3)-d-glucans and β-(1,6)-d-glucans. The interactions involved in the complexation process primarily include van der Waals forces and weak hydrogen bonds, indicating that the nature of these chemical interactions is similar to “adsorption” rather than “binding” [43].

##### Micro-Ionised Fibre

Micro-ionised fibres are derived from plant materials. They consist primarily of cellulose, hemicellulose, and lignin [25]. The fibre’s structural properties, such as surface area and pore size, play an essential role in determining their adsorption capacity. They undergo chemical interactions such as ion–dipole interactions, Van der Waals forces, and hydrogen bonding, which facilitate the adsorption of mycotoxins onto the fibre surfaces. Olive pomace, grape pomace, alfalfa hay, wheat straw, and grape stems have been used in several binding studies, and they exhibit a binding capacity that varies between 27% and 90%, based on the specific binder used and the type of mycotoxin involved [25]. Studies have shown that micronised wheat fibres can significantly lower the levels of AFB_1_ and OTA in animal tissues, including plasma and organs like the liver and kidneys [44]. It has also been reported that micronised wheat fibre can reduce OTA levels in the kidneys, liver and plasma of pigs [45]. Organic binders are better than inorganic binders at combating a broader spectrum of mycotoxins, making them particularly well-suited for addressing the common issue of multi-contamination in feeds and food commodities. Additionally, organic binders are biodegradable and do not remain in the environment with animal waste. In contrast, clays, which are often used in higher quantities than organic binders, might build up in manure and subsequently in fields during application, potentially causing harm to soils and pastures.

### 2.2. Applications of Conventional Binders in Aflatoxin Management

Conventional binders, including clays, activated carbon, yeasts, and bacterial cells, are commonly used to reduce aflatoxin absorption in contaminated foods and feeds by adsorbing aflatoxins in the gastrointestinal tract, thereby limiting toxin uptake and toxicity.

**Table 1:** Aflatoxin binders, mechanisms of action and their binding efficiencies.

Type of Binder	Applications	Mechanism of Action	Aflatoxin Binding Efficiency (%)	References
Activated Carbon	Detoxification of aflatoxin-contaminated animal feed	Adsorbs aflatoxins via a highly porous surface and micro/mesopores, trapping toxins physically	>99.5	[46]
Bentonite	Aflatoxin adsorption in livestock feed	Surface adsorption and ion exchange on layered aluminosilicate structure	86–98.5	[25,47]
Hydrated Sodium Calcium Aluminosilicate (HSCAS)	Reduces aflatoxin bioavailability in animals	Formation of stable inclusion complexes preventing absorption	83–100	[25,46]
Diatomite	Adsorption of aflatoxin in feed	High surface area adsorption via porous silica mineral structure	90–95	[46]
Esterified Glucomannan	Mycotoxin binding in gastrointestinal tract	Adsorption via polysaccharide chains that trap aflatoxins	96.6	[46]
Zeolite	Binding polar aflatoxin molecules in feed	Ion–exchange and adsorption on aluminosilicate framework	80	[46,48]
Montmorillonite	General adsorption of aflatoxins in animal feeds	Adsorption onto layered clay minerals, ion–exchange interactions	41	[46,47]
Yeast Cell Walls	Adsorption and reduction of aflatoxins in digestive tract	Binding of aflatoxins by polysaccharide components (glucans, mannans)	Moderate	[43,49]
Lactobacilli (Probiotics)	Biodegradation and binding of aflatoxins in feeds	Mycotoxin binding to bacterial cell walls and enzymatic degradation	Moderate	[50,51]
Sepiolite	Mycotoxin adsorption in animal feeds	Adsorption via fibrous clay mineral structure	95	[25]
Aluminosilicate	Multi-mycotoxin capture in feed	Adsorption and ion exchange on aluminosilicate clays	Variable	[25]

Toxin binders are primarily used in farm animals but also by humans [24]. Despite the extensive and successful application of the above strategies for AF control, limitations to their effectiveness still exist. The physical methods are usually less specific, and their effects can be reversed. In addition, the physical agents used, like clays, can bind important nutrients, thereby decreasing the nutrient content of food. The chemical agents used can produce toxic by-products and are not suitable for treating grains intended for food and feed purposes. Furthermore, the toxicity of by-products from enzymatic degradation has not yet been fully investigated [52]. There is a need for potential detoxifying agents that can neutralise AFs without affecting the nutritional value of food and feed upon exposure.

Researchers are investigating nanotechnology and plant-based solutions for new detoxifying agents, drawing inspiration from the long history of using plants as therapeutic agents. The integration of nanotechnology and traditional plant-based medicine, commonly known as nano-Ayurvedic medicine, is a promising approach to developing advanced therapeutic solutions. This innovative field makes use of nanotechnology to enhance the effectiveness and bioavailability of traditional Ayurvedic formulations that have been used for thousands of years, improving the delivery and efficacy of Ayurvedic treatments while reducing the risk of potential side effects.

## 3. Aflatoxin Mitigation Through Nanotechnology

Nanoparticles (NPs) are extremely tiny, sponge-like microscopic particles, roughly the size of a virus, containing several chambers that can be loaded with drugs [53,54]. Since they have a high surface area to volume ratio, they are used to load and move tiny molecules by trapping them in their internal cavities as a targeted delivery system [55]. Nanoparticles offer several advantages over traditional binders as they are capable of incorporating various bioactive compounds and have been shown to enhance the bioavailability of drugs [56].

### 3.1. Mechanisms of Action of Nanoparticles in Aflatoxin Control

Recent studies have highlighted the potent antifungal properties of NPs, which inhibit mold growth by inducing significant structural changes in fungal cell walls. Nanoparticles penetrate fungal cells primarily through adsorption and diffusion mechanisms. Adsorption involves NPs binding to negatively charged protein groups, disrupting proteins, and leading to cell death. At the same time, diffusion may produce reactive oxygen species (ROS) within cells, contributing to microbial destruction and inactivation [57,58]. They cause the formation of pits and pores in cell walls, compromising their integrity and disrupting the internal environment, which results in the leakage of essential cellular components [59]. Additionally, fungal cells cluster together, disrupting their normal growth patterns and reducing their ability to colonise new areas. The cell surface also undergoes shrinkage, impairing membrane functions and further inhibiting growth [59]. Nanoparticles can also induce external and internal distortions, altering the structures of internal organelles such as the mitochondria, which are crucial for cellular function. Nanoparticles can also generate ROS, which damage macromolecular structures within fungal cells. This oxidative stress effectively blocks enzyme active sites and inhibits metabolic processes, resulting in cell death [60]. Another way in which NPs function is through phagocytosis, triggered by opsonins like complement proteins and immunoglobulin-like antibodies, which attach to the NP surface, allowing phagocytes to identify and bind the NPs through specific ligand–receptor interactions. This triggers a series of signalling processes that lead to the build-up of actin, formation of cell extensions, and engulfment of the NPs, resulting in the formation of a phagosome [60].

The antifungal potential of NPs combines the physical disruption of cell structures with biochemical interference in cellular metabolism. This dual mechanism highlights the efficacy of NPs as antifungal agents, offering a promising alternative for managing fungal infections.

### 3.2. Green Nanotechnology

Green nanotechnology is a field of research pioneered by Katti et al. [61] that integrates the principles of green engineering and green chemistry to develop environmentally friendly nanomaterials and nanoproducts. This process utilises plant-based materials and other biomaterials, such as bacteria, yeasts, and fungi, as electron-rich chemical reducing and stabilising agents to convert metal precursors into various functionalised NPs [61,62]. These beneficial bioactive compounds are obtained from sources such as leaves, stems, roots, seeds, fruits, tree bark, and gums [63]. The therapeutic effectiveness of herbal medicinal plants relies on the bioactive phytochemical compounds they contain, like alkaloids, carotenoids, flavonoids, phenolics, tannins, glycosides, quinones, terpenoids, saponins, thiosulfinates, glucosinolates and organic acids [64]. So far, about 12,000 different phytochemicals from plants have been identified and characterised, representing less than 10% of the total phytochemicals that exist [65]. However, many phytochemicals have poor solubility, leading to limited bioavailability and faster clearance from the body [66]. As a result, higher doses of herbal materials are often required to achieve therapeutic effects, which can make their practical use challenging. To address these issues related to plant medicines, specifically low solubility and bioavailability, integrating bioactive plant components with nano-sized drug delivery systems (NDDS) can be a practical solution.

### 3.3. Bioactive Plant Phytochemicals and Their Role in Green-Synthesis of Nanoparticles

Pharmaceutical formulations derived from bioactive plant sources have made a substantial impact on the treatment of diseases [67]. Over 80% of the global population depends on medicinal plants for the treatment of various diseases, such as cancer, tuberculosis, diabetes, and high blood pressure [68]. The dependency on herbal remedies is most significant in many developing countries, where access to conventional medicine may be limited [69,70]. They can enhance the clinical effectiveness of conventional antibiotics by increasing their potency, hence contributing to the reduction of antibiotic resistance [71]. The global market for herbal medicine continues to grow as more people seek natural alternatives to synthetic drugs [72,73]. This is driven by factors such as the rising cost of healthcare, increasing awareness of the side effects associated with conventional medications, and a growing interest in holistic health approaches.

Polyphenolic compounds, including phenols and flavonoids, are vital components of plants known for their significant antioxidant activity. These compounds exhibit numerous biological activities, such as antimicrobial, biosorbent, and anti-cancer effects [74]. Some of these bioactive compounds have been used as biosorbent additives in food and feed as a measure for limiting the effects due to exposure to AFs. These biosorbent additives not only act as biosorbents in food and feed, but they also improve the therapeutic value of these commodities.

Plant phytochemicals play an important role in green nanoparticle synthesis because they act as natural reducing and stabilising agents. These bioactive compounds, such as phenolic compounds, terpenoids, flavonoids, proteins, and other antioxidants found in plant extracts, convert metal ions such as Ag^+^, Au^3+^, and Cu^2+^ into their corresponding metal nanoparticles while also preventing aggregation by capping and stabilising the particles [75]. This allows for the rapid creation of nanoparticles with controlled size, shape, and increased stability without the need for harsh chemical inputs.

### 3.4. Green-Synthesised Nanoparticles as Biosorbents

Green synthesis of NPs can be achieved through three primary methods: physical, chemical, and biological [76]. Physical methods involve techniques like evaporation–condensation and laser ablation, which produce high-purity NPs but often suffer from low yields and high energy consumption. Chemical methods use organic and inorganic reducing agents like sodium borohydride (NaBH_4_) and capping agents like polyvinylpyrrolidone (PVP) [77]. They produce high yields and control over particle properties, but potentially generate hazardous by-products. The biological method is a plant-based and microbial synthesis method. The biological method offers a range of advantages over the chemical and physical synthesis methods. It is more environmentally friendly since non-toxic reagents, which are eco-friendly and safe, are used. It is also pollution-free, economical, more sustainable, less time-consuming, and produces biocompatible products [78,79].

Metallic NPs are widely used as antimicrobial agents since they have a high surface area-to-volume ratio, greater stability, and biocompatibility. They possess a high surface charge and can exist in both crystalline and amorphous structures, with sizes that range from 10 nm to 100 nm. Frequently used metals include silver, gold, copper, iron, cobalt, zinc, magnesium, platinum, etc., and have been found to possess beneficial health effects [80,81]. Gold NPs (AuNPs) offer a variety of applications in diagnostics and drug delivery thanks to their distinct features. They are widely researched for use in medicinal and Ayurvedic formulations in India and China. Silver NPs (AgNPs) are utilised in various biomedical fields, such as separation science and advanced drug delivery systems, due to their anti-inflammatory and antimicrobial properties [81]. Previous research has demonstrated that herbal extracts have hepatoprotective effects by reducing oxidative stress induced by toxins and other toxic components [82]. The selection of plant species for nanoparticle (NP) synthesis is based on their historical use in traditional medicine and their phytochemical content, which influences their antioxidant, antimicrobial, and anti-inflammatory properties, alongside their ability to reduce the chosen metal precursor [83].

**Figure 3:**
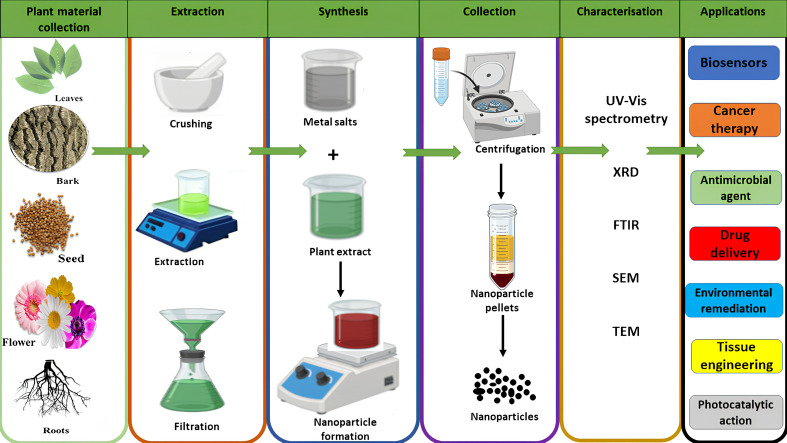
Illustration of plant-mediated metal nanoparticle synthesis.

The process of synthesising NPs (Figure 3) from plant materials begins with carefully selected plant components, such as leaves, bark, roots, seeds, fruits, or fruit peels. These materials are collected and thoroughly rinsed with distilled water to eliminate any surface contaminants and then crushed. For the extraction process, solvents like distilled water, methanol, ethanol, or acetone are added to the crushed plant material and heated to promote the release of bioactive compounds into the solvent. Following this step, the mixture is filtered to obtain a clear plant extract. To evaluate the phytochemical content of this extract, mass spectrometry is employed [84]. Subsequently, the prepared plant extract is combined with a metal salt solution at specific concentrations determined by the chosen methodology while stirring continuously. During this reaction, bioactive phytochemicals present in the extract function as both reducing and capping agents, enabling the reduction of metallic salts into metal NPs [85,86,87]. Once synthesised, the metallic nanoparticles undergo characterisation using various techniques such as UV–Visible spectrometry, Fourier Transform Infrared Spectroscopy (FTIR), Transmission Electron Microscopy (TEM), X-ray Diffraction (XRD), and Scanning Electron Microscopy (SEM). These methods allow researchers to assess the absorbance, composition, shape, size, and surface charge of the NPs, which are critical elements in determining their efficacy for specific applications.

While there is growing interest in using green-synthesised metal NPs for various applications, including antimicrobial and antifungal purposes, the specific use of these NPs in aflatoxin decontamination and detoxification remains relatively underexplored. However, existing studies (Table 2) suggest promising potential for green-synthesised NPs in this area.

## 4. Encapsulation of Green-Synthesised Nanoparticles Using β-Cyclodextrin for Enhanced Aflatoxin Detoxification/Degradation

**Figure 4:**
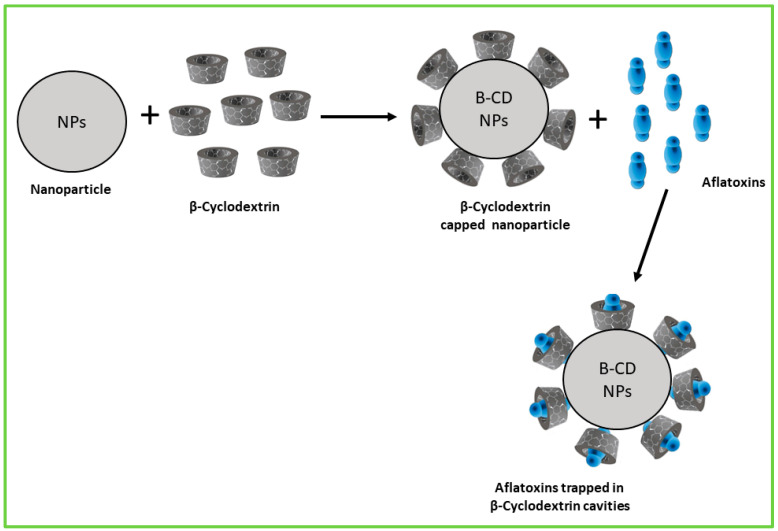
Schematic representation of encapsulation of NPs using β-CD for enhanced aflatoxin adsorption.

Encapsulation of green-synthesised NPs using β-CD offers a powerful approach to enhance their stability, solubility, and controlled release, thereby improving the efficiency of aflatoxin detoxification and degradation (Figure 4). β-CD’s unique molecular structure enables it to form inclusion complexes by trapping hydrophobic NPs within its hydrophobic cavity, protecting the NPs from aggregation and environmental degradation while increasing their bioavailability and sustained activity. This encapsulation strategy holds promise for developing safer and more functional nanomaterials for aflatoxin management in food and feed systems.

β-Cyclodextrins (β-CD) and their derivatives are a novel class of sorbents that have gained significant scientific interest from researchers in both academia and industry due to their typical chemical, structural, and physical properties [99]. These unique properties make them more versatile agents for removing targeted metabolites in various applications, including environmental remediation, pharmaceutical formulations, and food safety. These properties include the following:

• Toroidal-shaped Cavity Structure that allows them to encapsulate guest molecules, through host–guest interactions, accommodating molecules of appropriate size and shape [100].
• Hydrophilic Outer Surface, which facilitates interactions with water molecules and enhances the solubility of the cyclodextrin–metabolite complex.
• Selective binding capacity towards certain molecules based on their size, shape, and polarity, which allows it to specifically target and remove toxic molecules from a mixture of compounds [101].
• Biocompatibility, making it suitable for various biomedical and pharmaceutical applications [102].
• Thermal Stability to withstand a wide range of environmental conditions [103].

### 4.1. Methods Used for Nanoencapsulation

#### 4.1.1. Co-Precipitation

The nanomaterial is dissolved in a small amount of ethanol at 60 °C, then added dropwise into a conical flask containing a β-CD solution with vigorous stirring and heating to evaporate all the ethanol. It is then cooled and stored at 4 °C overnight, filtered and dried in a vacuum oven [104].

#### 4.1.2. Solvent Evaporation

Appropriate amounts of β-CD and nanomaterials are dissolved in a volatile solvent like methanol and stirred until the solution runs clear. The solvent is evaporated using a rotary vacuum evaporator and followed by drying in a vacuum dryer until all the solvent is completely evaporated. The dried sample is passed through a mesh sieve and then stored until further evaluation [105].

#### 4.1.3. Kneading

β-cyclodextrin and the nanomaterial are blended in a mortar with a small quantity of alcohol and distilled water to obtain a slurry-like consistency. The paste is dried in an oven, then pulverised and passed through a fine sieve to obtain a powdered complex [104].

#### 4.1.4. Freeze-Drying

In this method, β-CD is dissolved in distilled water and stirred until the solution is clear. The desired nanomaterials for encapsulation are added and stirred. The reaction mixture if then filtered and the filtrate freeze-dried to obtain a solid complex which can be ground and kept for characterisation [105].

#### 4.1.5. Spray Drying

With this method, a solution of β-CD and the nanoparticle material is sprayed into a heated chamber, where the solvent evaporates, and the encapsulated NPs are collected [106].

Other methods include microwave irradiation, ionic gelation, sub-critical carbon dioxide and grinding. Successful encapsulation can be confirmed by several characterisation techniques such as UV–Vis spectrometry, FTIR and SEM [107]. The selection of a β-CD nanoencapsulation method depends on the guest molecule’s properties, desired encapsulation efficiency, particle size, scalability, cost, and safety. Milder methods are preferred for sensitive compounds, while techniques offering uniform nanoscale particles enhance bioavailability. Simpler, cost-effective methods like grinding or co-precipitation are easier to scale, and food-safe, solvent-free processes are favored due to regulatory and safety concerns. Maintaining the stability of the active compound is also essential for successful encapsulation.

### 4.2. Limitations, Regulatory and Safety Concerns in the Use of Green Nanotechnology for Aflatoxin Management

Green-synthesised NPs offer a promising eco-friendly approach to aflatoxin mitigation by leveraging their antioxidant properties and high aflatoxin adsorption capacity to neutralise toxins and inhibit fungal growth. However, practical application of nanotechnology in aflatoxin management faces several significant limitations that hinder its widespread adoption.

#### 4.2.1. Stability and Cost-Effectiveness

Stability challenges caused by variations in synthesis methods and biomolecules might impact nanoparticle consistency and shelf life, limiting their long-term performance [108]. Furthermore, scaling up production from laboratory to industrial levels remains challenging and expensive, as procedures designed for small batches may not translate well to large-scale manufacture [109].

#### 4.2.2. Regulatory Hurdles

Regulations continue to pose major challenges, due to the lack of integrated, globally approved guidelines specifically adapted for green nanotechnology applications in agriculture and food. Currently, regulatory systems vary greatly between countries, with many lacking clear guidelines for assessing the unique features and dangers associated with nanomaterials manufactured using green technologies [110]. Challenges in regulation stem from the difficulties in defining nanomaterials consistently, developing standardised testing and characterisation protocols, and managing diverse data requirements, especially for global market access. Regulatory bodies prioritise comprehensive physicochemical characterisation, manufacturing quality, and robust safety and efficacy data, including assessments of long-term toxicity and environmental impact.

The Organisation for Economic Co-operation and Development (OECD) Test Guidelines Programme (TGP) is actively developing and updating several Test Guidelines focused on the physicochemical and environmental properties of nanomaterials, such as particle size, surface area, dissolution rate in aquatic media, and removal from wastewater. Alongside developing regulatory testing methods, new tools for assessing nanomaterial hazards and exposures are emerging. The OECD highlights the importance of standardised data collection and notes that creating test guidelines for nanomaterials requires extensive research and validation, making the process time and resource-intensive. Alternative approaches such as in vitro assays, high-throughput screening (HTS), and quantitative structure–activity relationships (QSARs) show strong potential for evaluating nanomaterial safety, although they have yet to be officially incorporated into OECD testing guidelines [111].

#### 4.2.3. Impact on Human and Animal Health

Nanoparticles are very small and highly reactive, allowing them to pass through natural barriers in the body like cell walls and the blood–brain barrier, which usually block harmful substances [112]. Since they can reach and build up in places they should not, there are concerns about their safety. Once inside, they may cause harmful effects such as inflammation, damage to cells from unstable molecules called free radicals, or changes to DNA that can lead to mutations and other problems. Previous studies have found that carbon-based NPs cause size-dependent toxicity in lung cancer cells [113]. Individual diversity in reaction to NPs complicates safety assessments due to differences in genetic makeup, immune system function, metabolism, and overall health status. These differences can influence how NPs are absorbed, transported, metabolised, and eliminated by the body, resulting in varying susceptibility to possible harmful effects [110]. Additionally, pre-existing health issues, such as respiratory diseases, allergies, or immunological disorders, might influence how the body reacts to nanoparticle exposure. As a result, traditional toxicity studies that rely on average responses may fail to capture these subtle effects, making it difficult to establish universally protective exposure limits.

#### 4.2.4. Impact on the Environment

Nanoparticles, due to their small size and great mobility, can move more easily across ecosystems than larger particles, potentially endangering plant and animal health. According to research, certain NPs can accumulate in natural habitats, potentially affecting ecological systems [114]. As a result, it is critical to thoroughly assess their environmental impact and develop safer alternatives, such as biodegradable or less toxic NPs, to minimise potential harm.

Although the potential to improve food quality and agricultural productivity is evident, it is essential to systematically address concerns related to safety, environmental impact, and regulatory complexities. Addressing these limitations requires a comprehensive, multifaceted approach. Standardising synthesis protocols alongside the development of innovative encapsulation techniques can significantly enhance nanoparticle stability and functionality. Investing in scalable and environmentally sustainable production methods will facilitate large-scale deployment while maintaining cost-effectiveness. To address these regulatory hurdles, international cooperative efforts, such as those led by the OECD, are underway to standardise testing guidelines, improve data acceptance, and harmonise regulations across jurisdictions [111]. Close collaboration with regulatory agencies, coupled with the generation of robust safety and efficacy data, is essential to secure regulatory approval and build consumer trust. Moreover, thorough toxicological evaluations that consider individual population variability are critical for anticipating long-term impacts on human health and the environment. The advancement of less toxic, biodegradable nanoparticle alternatives, together with strict quality control practices, will further ensure the safe and responsible integration of nanotechnology into aflatoxin management.

## 5. Conclusions and Future Perspectives

Aflatoxins remain a major global issue due to their widespread occurrence, grievous health impacts, and economic burdens. Chronic exposure to AFs has been linked to hepatocellular carcinoma, immunosuppression, and stunted growth in children, disproportionately affecting populations in underdeveloped nations with limited regulatory enforcement and awareness. Current AF management strategies focus on preventing fungal infestation through good agricultural practices, improved storage, and biological, physical, and chemical control measures. However, if contamination still occurs, post-harvest decontamination and detoxification measures are necessary to reduce the impact of AF contamination.

Green-synthesised NPs considerably improve the stability and bioavailability of volatile plant compounds while enhancing environmental sustainability. Previous research has shown that NPs can form inclusion complexes with β-CD, which enhances the solubility and thermal stability of the NPs and prevents their disintegration when subjected to environmental factors such as changes in pH and light. Encapsulation of NPs with β-CD also improves the binding efficiency and provides additional bioactive properties from the incorporated phytochemicals that could lead to AF degradation.

Conventional binders such as inorganic clays, activated carbon, and organic binders, while efficient, have some drawbacks, including fluctuating adsorption capacity, unfavorable interactions with nutrients, and an adverse effect on the environment. The shift from conventional binders to greener solutions is imperative as it offers a more sustainable approach to aflatoxin management while addressing the urgent need for more effective, eco-friendly and safer aflatoxin management strategies to ensure food safety.

## Figures and Tables

**Table 2:** Studies evaluating the role of green synthesised NPs in aflatoxin mitigation.

Biogenic Extract	Type of Nanoparticle	Study Model	Aflatoxin Inhibition (%)	References
*Syzgium cumini* (Leaf extract)	AgNPs	In vitro	100% inhibition of AF production	
Green tea and black tea (leaf extract)	FeONPs	Peanut puree	43.7% and 41.2% reduction of AFB_1_ and AFB_2_, respectively	[88]
*Curcuma longa* L. (rhizome)	AgNPs	In vitro on spiked broiler feed samples	100% inhibition of mycelia growth	[89]
*Pleurotus ostreatus* (mushroom substrate extract)	ZnO-CuONPs	In vitro on potatoes dextrose agar (PDA)	13.1% inhibitory effect on *A. flavus* on media with 0.5 ppm concentration	[90]
*Juglans-regia* (leaf extract)	AgNPs	In vivo using albino mice	Reduction of kidney and liver injury induced by AFB_1_	[91]
*Cissus quadrangularis*	CuONPs	In vitro on PDA broth	83% and 86% inhibition of *A. niger* at 500 ppm and 1000 ppm respectively. *A. flavus* 81% and 85% inhibition at 500 ppm and 1000 ppm, respectively	[92]
*Beta vulgaris*	ZnONPs	In vitro on Mueller–Hinton agar medium	Growth inhibition	[93]
*Azadirachta indica* (leaf extract)	Fe/MgNPs	In vitro by agar well diffusion assay	Growth inhibition of *A. flavus*	[94]
*Bacillus subtilis* (bacteria)	AgNPs	In vitro assay on peanut sample	Reduced AF production by 82.53%	[95]
Green tea and black tea (leaf extracts)	Ag, Fe, CuNPs	In vitro	AF reduction	[96]
*Penicillium verrucosum* (fungi)	AgNPs	In vitro on PDA	Suppressed growth of *A. flavus* by 50%	[97]
Pomegranate (peel extract)	AgNPs	In vivo on broiler chicks	Prevention of AF-induced pathologies. Reduced AF concentration from 1.91 ppm to 1.85 ppm	[98]

## Data Availability

No data was used for the research described in the article.
